# Accelerating evidence synthesis for safety assessment through ClinicalTrials.gov platform: a feasibility study

**DOI:** 10.1186/s12874-024-02225-2

**Published:** 2024-07-30

**Authors:** Tianqi Yu, Xi Yang, Justin Clark, Lifeng Lin, Luis Furuya-Kanamori, Chang Xu

**Affiliations:** 1 Center of Research in Epidemiology and Statistics, Université Paris Cité, Inserm, Paris, France; 2 Department of Maternal, Child and Adolescent Health, School of Public Health, Anhui Medical University, Hefei, Anhui China; 3 Institute for Evidence-Based Healthcare, Faculty of Health Sciences and Medicine, Bond University, Gold Coast, QLD Australia; 4 Department of Epidemiology and Biostatistics, University of Arizona, Tucson, AZ USA; 5 UQ Centre for Clinical Research, The University of Queensland, Herston, Australia; 6 Proof of Concept Center, Eastern Hepatobiliary Surgery Hospital, Third Affiliated Hospital, Second Military Medical University, Naval Medical University, Shanghai, China

**Keywords:** Evidence synthesis, Safety assessment, ClinicalTrials.gov, Empirical investigation

## Abstract

**Background:**

Standard systematic review can be labor-intensive and time-consuming meaning that it can be difficult to provide timely evidence when there is an urgent public health emergency such as a pandemic. The ClinicalTrials.gov provides a promising way to accelerate evidence production.

**Methods:**

We conducted a search on PubMed to gather systematic reviews containing a minimum of 5 studies focused on safety aspects derived from randomized controlled trials (RCTs) of pharmacological interventions, aiming to establish a real-world dataset. The registration information of each trial from eligible reviews was further collected and verified. The meta-analytic data were then re-analyzed by using 1) the full meta-analytic data with all trials and 2) emulated rapid data with trials that had been registered and posted results on ClinicalTrials.gov, under the same synthesis methods. The effect estimates of the full meta-analysis and rapid meta-analysis were then compared.

**Results:**

The real-world dataset comprises 558 meta-analyses. Among them, 56 (10.0%) meta-analyses included RCTs that were not registered in ClinicalTrials.gov. For the remaining 502 meta-analyses, the median percentage of RCTs registered within each meta-analysis is 70.1% (interquartile range: 33.3% to 88.9%). Under a 20% bias threshold, rapid meta-analyses conducted through ClinicalTrials.gov achieved accurate point estimates ranging from 77.4% (using the MH model) to 83.1% (using the GLMM model); 91.0% to 95.3% of these analyses accurately predicted the direction of effects.

**Conclusions:**

Utilizing the ClinicalTrials.gov platform for safety assessment with a minimum of 5 RCTs holds significant potential for accelerating evidence synthesis to support urgent decision-making.

**Supplementary Information:**

The online version contains supplementary material available at 10.1186/s12874-024-02225-2.

## Background

In clinical research, evaluating interventions involves assessing both effectiveness and safety, which are crucial considerations [1]. However, obtaining conclusive evidence regarding safety can be challenging due to sparse data, which are prone to random errors and often lack statistical power [2–5]. Systematic reviews with meta-analysis offer a feasible solution to this challenge [6]. By pooling information from multiple trials on the same topic, it enhances statistical power and reduce uncertainty, thereby enabling more robust and informed conclusions [7].

Nevertheless, conducting a standard systematic review can be labor-intensive and time-consuming. It has been estimated that producing a high-quality systematic review may take between 6 months to 2 years [8]. This presents a challenge, particularly in situations where decision-makers require timely evidence to inform urgent decisions and response measures.

To expedite the evidence synthesis process [9], two distinct approaches have been proposed. The first approach involves leveraging automation tools or software equipped with machine learning or deep learning algorithms to aid in evidence synthesis tasks such as screening and data extraction [10]. Another is the rapid review approach, which streamlines the standard systematic review processes to efficiently generate evidence [11], such as assigning a single reviewer in each step while another reviewer verifies the results, excluding or limiting the use of grey literature, or by narrowing the scope of the review.

A common strategy employed in rapid reviews involves gathering potential evidence from selected databases rather than all available ones. Among the various databases of trials records, ClinicalTrials.gov (https://clinicaltrials.gov/) emerges as a promising option. This is primarily because it is the world's largest trial repository of results data from clinical trials. It can often provide much of the information required format extraction in systematic reviews. Moreover, unlike traditional publications, the data presented on ClinicalTrials.gov are often well-structured into multiple tables, enhancing readability and facilitating the extraction of target values. This structured format also lends itself to automated processes, potentially making evidence synthesis more efficient [12].

Pradhan et al. pioneered the development of an automated data extraction tool designed to generate analysis-ready spreadsheets containing trial data sourced from ClinicalTrials.gov [13]. Employing this tool, they replicated meta-analyses conducted in three previously published systematic reviews, revealing that 83.3% of the published estimates could be replicated using data exclusively from ClinicalTrials.gov [13]. However, relying solely on data from ClinicalTrials.gov for accuracy estimates, as demonstrated in just three published systematic reviews, may not provide sufficient evidence to validate its efficacy against standard systematic review methods.

In this study, we aim to thoroughly investigate the potential of rapid review through ClinicalTrials.gov using a large-scale empirical dataset. Specifically, we conduct an in-depth comparison of the point estimates and effect obtained from meta-analyses conducted using data from ClinicalTrials.gov with those derived from standard systematic reviews.

## Methods

### The empirical dataset

A search was conducted on PubMed for systematic reviews focusing exclusively on adverse events from January 1, 2015, to January 1, 2020 (refer to the Supplementary file for the search strategy), which forms the basis of current empirical dataset. Adverse events were defined as ‘*any untoward medical occurrence in a patient or subject in clinical practice*’ [14]. The representativeness of the search has been well-defined, with a sensitivity of 93.9% [15]. Systematic reviews addressing pharmacological interventions with at least one meta-analysis with at least 5 randomized controlled trials (RCTs) and providing 2 by 2 table data for each included study of the meta-analysis were deemed eligible. Non-pharmacological interventions (e.g., surgical or device interventions) were excluded. These interventions often involve a combination of treatments, including medications and nursing care, making it challenging to pinpoint the specific adverse events they may cause. Further selection criteria involved limiting systematic reviews to those based on randomized controlled trials (RCTs). This decision was based on that RCTs are more likely to be registered compared to other study types, with well-conducted systematic reviews of RCTs anticipated to offer the highest level of evidence [16].

The metadata (2 by 2 table data for each study per meta-analysis) from each systematic review were collected from forest plots or baseline characteristic tables. This process involved four independent data extractors, with subsequent double-checking to ensure accuracy. To minimize variability in point estimates stemming from random error, only meta-analyses with five or more included studies were considered during data extraction [17]. Additionally, the validity of the 2 by 2 table data for each study within these meta-analyses was verified by consulting original sources, such as the original publications of the RCTs, supplementary files, registration platforms, and company websites. Any potential data extraction errors identified during this phase were documented and rectified to uphold data quality standards. A detailed account of this process is available elsewhere [15].

#### Emulating rapid meta-analyses

To simulate rapid meta-analyses, all studies within each meta-analysis were examined for registration information. Studies registered on ClinicalTrials.gov and posting identical results to the empirical dataset formed the dataset for rapid meta-analysis. For instance, if a meta-analysis in the empirical dataset comprised 10 RCTs, of which 7 were registered on ClinicalTrials.gov and had posted identical results, then data synthesis for the full meta-analysis was conducted using all 10 RCTs, while data synthesis for the rapid meta-analysis utilized the 7 RCTs registered on ClinicalTrials.gov. Studies that were registered but did not post their results on ClinicalTrials.gov or were registered on other platforms were excluded from the rapid meta-analysis.

To identify the studies to include in the rapid meta-analysis, the following steps were followed. Initially, we attempted to retrieve the NCT number from the original publication of each study. Subsequently, we conducted a search on ClinicalTrials.gov using the NCT number to ascertain whether the identifiers matched those of the original studies and to verify if the summarized results were posted. This process was performed by one review author (TQ) and subsequently double-checked by another (CX).

#### Missing data

If the original sources (i.e., publications, registry records, etc.) of the included studies could not be obtained, it would be impossible to collect the registration information. In such cases, given the anticipated small proportion of missing data, studies without accessible original sources were not included in the rapid meta-analysis. This approach may result in an underestimation of the performance of rapid meta-analysis.

#### Statistical analysis

Both the full meta-analyses and rapid meta-analyses underwent re-analysis using the same methods. Given that safety outcomes often include zero-events studies, we employed the Mantel–Haenszel (MH) method and random slope generalized linear mixed model (GLMM) – recognized as two-stage and one-stage approaches, respectively – to address the zero-events issue more effectively [18]. To account for potential heterogeneity across studies, both fixed and random MH models were utilized. The odds ratio (OR) with a 95% confidence interval (CI) served as the effect estimator [19].

In certain meta-analyses, trial data were used more than once due to the inclusion of multiple interventions or different doses (e.g., 10 mg vs. placebo, 5 mg vs. placebo), resulting in a single trial being erroneously treated as two or more trials. Consequently, this duplication led to the repeated utilization of the control sample size, yielding overconfident estimates. To address this issue, we retained only the first comparison for each trial in the meta-analysis, ensuring that each trial was used only once to avoid redundant utilization of information.

The comparison between rapid meta-analysis and full meta-analysis involved assessing both the magnitude and direction of the estimated pooled odds ratio (OR). Magnitude comparisons were based on predefined difference cutoff points, set at 5%, 10%, 15%, and 20% [20], representing tolerable bias thresholds. The tolerable bias was calculated as $\left|\widehat{\theta }-{\widehat{\theta }}_{r}\right|/\widehat{\theta }$, where $\widehat{\theta }$ was the point estimate for the full meta-analysis and ${\widehat{\theta }}_{r}$ was the point estimate for the emulated rapid meta-analysis.

If the bias fell below these cutoff points, the rapid meta-analysis was considered to have achieved precise effect estimates. Regarding direction, changes were categorized as either beneficial effects to harmful effects or harmful effects to beneficial effects in the rapid versus full meta-analysis comparison. Additionally, the significance of *P*-values was compared across both analyses, encompassing total changes, shifts from significance to non-significance, and shifts from non-significance to significance.

Given the potential influence of event rates and heterogeneity on the results, subgroup analyses were conducted based on: 1) event rates stratified by percentiles (25th, 50th, and 75th); and 2) levels of heterogeneity, assessed using I-squared and categorized as ≤ 25% (indicating no or mild heterogeneity) and > 25% (reflecting moderate to large heterogeneity) [21]. All analyses were executed using the R program (version 4.1.3), with a significance level set at α = 0.05.

## Results

Out of 18,636 records from PubMed screened, 456 systematic reviews focusing on adverse events were identified. Among them, 151 systematic reviews met our inclusion criteria. A detailed screening process was documented in our previous publications [15, 22]. From these 151 eligible systematic reviews, a total of 611 meta-analyses comprising at least five studies were identified, with a combined total of 7,605 RCTs. This dataset serves as the primary dataset for the present study (refer to Figure S1). Registration data were missing in 256 (3.37%) RCTs.

During the validation of data accuracy, we identified 1,497 RCTs with data extraction errors as reported by the original authors of the systematic reviews. Among these errors, 457 instances (30.5% of the total errors) could not be rectified due to the unclear definition of safety outcomes in the systematic reviews. In order to mitigate potential bias resulting from these ambiguous errors, we excluded these 457 RCTs from the original meta-analyses. Additionally, RCTs that were incorrectly included in the meta-analyses (e.g., 2 RCTs were included although they reported data from a single trial) were also excluded based on a post hoc decision, amounting to a total of 114 RCTs. Following these exclusions, 558 meta-analyses (comprising 91.3% of the initial set) still contained at least five studies and thus remained eligible for analysis. The exclusion of 53 meta-analyses involving 571 RCTs did not significantly change the baseline characteristics, as shown in Table 1.

**Table 1:** Baseline characteristics of the dataset for analysis

Baseline characteristics	Original dataset (*n* = 611)	Error addressed dataset (*n* = 558)	Dataset for analysis (*n* = 502)
Number of studies per meta-analysis (Median, Q1 to Q3)	9 (6 to 13)	9 (6 to 14)	9 (7 to 14)
Total sample size per meta-analysis (Median, Q1 to Q3)	4,419 (2,380 to 7,881)	4,373 (2,546 to 7,842)	4,758.5 (2,713 to 8,176)
Registration rate on all platforms per meta-analysis (Median, Q1 to Q3)	85.7% (48.1% to 100%)	86.5% (47.4% to 100%)	88.6% (62.5% to 100%)
Registration rate on ClinicalTrials.gov per meta-analysis (Median, Q1 to Q3)	81.2% (37.5% to 100%)	83.3% (37.5% to 100%)	87.1% (50% to 100%)
Registered with posed results on ClinicalTrials.gov per meta-analysis (Median, Q1 to Q3)	63.6% (16.9% to 85.7%)	63.6% (18.2% to 87.0%)	70.1% (33.3% to 88.9%)

For the error addressed dataset, there were 56 (10.0%) meta-analyses with none of the studies registered and therefore these were not included in the analysis dataset. The registrations of the RCTs were identified based on the reporting of the NCT number in the original sources of the RCTs

### Trial registration characteristics

Among the 558 meta-analyses, the median number of studies per meta-analysis was 9 (interquartile range [IQR]: 6 to 13), and the median sample size of each meta-analysis was 4,373 (IQR: 2,546 to 7,842). The percentage of RCTs registered on ClinicalTrials.gov within each meta-analysis varied from 0 to 100%, with a median value of 83.3% (IQR: 37.5% to 100%). Moreover, the proportion of RCTs registered on ClinicalTrials.gov and with posted results ranged from 0 to 100%, with a median value of 63.6% (IQR: 18.2% to 87.0%). Notably, there were 56 meta-analyses (10.0%) where all included RCTs were unregistered and thus unsuitable for rapid synthesis. For the remaining 502 meta-analyses utilized to emulate rapid meta-analysis, the median number of studies per meta-analysis was 9 (IQR: 7 to 14), with a median proportion of 70.1% (IQR: 33.3% to 88.9%) of the RCTs registered on ClinicalTrials.gov and with posted safety results (see Table 1).

### Rapid synthesis using RCTs registered in ClinicalTrials.gov

Due to convergence issues and the “sparse data bias” commonly encountered by the GLMM model [23], 100 cases (19.9%) failed to attain valid estimations for either full or rapid meta-analyses across the entire dataset. Meanwhile, under the MH model, 12 cases (2.4%) were unable to achieve valid estimations due to zero-event issues. Consequently, these cases were excluded from the subsequent analysis.

**Fig. 1:**
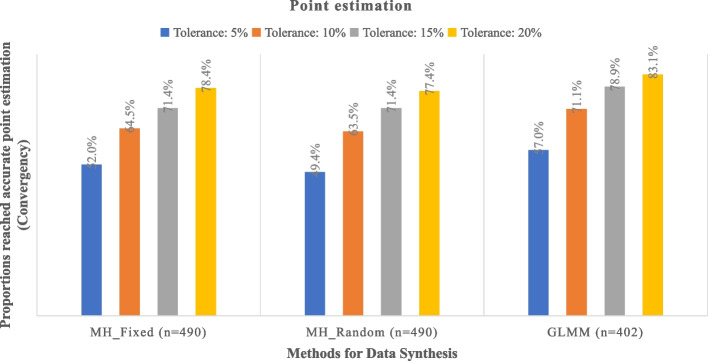
Consistency regarding the point estimates of rapid approach through ClinicalTrials.gov and systematic approach

Figure 1 illustrates the bias observed in rapid meta-analyses compared to full meta-analyses. Regarding effect magnitude, within the tolerance thresholds of 5%, 10%, 15%, and 20%, between 49.4% and 57.0%, 63.5% and 71.1%, 71.4% and 78.9%, and 77.4% and 83.1% of rapid meta-analyses were able to produce acceptable point estimates. Notably, the GLMM model demonstrated the highest proportion of achieving acceptable point estimates (ranging from 57.0% to 83.1%).

**Fig. 2:**
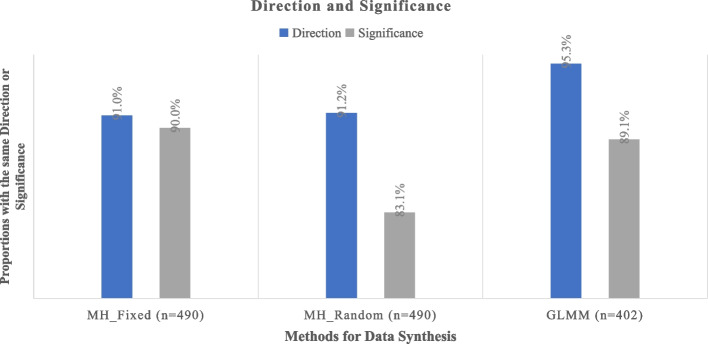
Consistency regarding the direction and significance *P*-value of rapid approach through ClinicalTrials.gov and systematic approach

Regarding the direction of effects, between 91.0% and 95.3% of rapid meta-analyses exhibited consistent effect directions compared to full meta-analyses. Notably, the GLMM model demonstrated the highest consistency among the three methods. In cases where rapid meta-analyses diverged in direction from full meta-analyses, between 45.5% (fixed MH) and 81.0% (GLMM) indicated beneficial effects in rapid meta-analyses but harmful effects in full meta-analyses, while between 19.0% (GLMM) and 54.5% (fixed MH) showed harmful effects in rapid meta-analyses but beneficial effects in full meta-analyses (refer to Fig. 2).

Regarding significance, approximately 83.1% to 90.0% of rapid meta-analyses maintained consistent significance levels of *p*-values compared to full meta-analyses. Notably, the fixed MH model exhibited the most robust performance among the three methods. In cases where rapid meta-analyses diverged in significance from full meta-analyses, between 18.4% (fixed MH) and 20.5% (GLMM) showed significant *p*-values in rapid meta-analyses but non-significant *p*-values in full meta-analyses, while between 79.5% (GLMM) and 81.6% (fixed MH) exhibited non-significant *p*-values in rapid meta-analyses but significant *p*-values in full meta-analyses (refer to Fig. 2).

### Subgroup analysis

Subgroup analysis was conducted based on event rates and heterogeneity, utilizing predefined intervals as outlined in the Methods section. Concerning event rates, outcomes with lower incidence rates demonstrated more consistent point estimates and significance levels of *p*-values between rapid meta-analysis and full meta-analysis compared to outcomes with higher incidence rates (see Figures S2-S4). Regarding heterogeneity, higher levels of heterogeneity tended to result in discordant findings between rapid meta-analysis and full meta-analyses, owing to biases in magnitude and significance of *p*-values. However, our study found that meta-analyses with null or mild heterogeneity exhibited a higher proportion of discordant effect directions between rapid meta-analysis and full meta-analyses (see Figures S5-S7).

#### Example

We utilized the systematic review conducted by Thomas et al. as a case study [24]. The data utilized in their systematic review were confirmed to be error-free, except for a typographical error in a forest plot. The focus of their investigation was the neuropsychiatric adverse events associated with varenicline, with suicidal ideation events as the primary outcome, which we adopted for our analysis. Among the 20 RCTs included in their review, 14 were found to be registered on ClinicalTrials.gov (Supplementary Table 1). Out of these 14 registered RCTs, results were posted on ClinicalTrials.gov for 11, forming the dataset for rapid synthesis. Employing the same method (Peto’s OR) as used in the original review, we obtained a pooled OR of 0.58 (95% CI: 0.28 to 1.20; *P* = 0.14) for the full meta-analysis and a pooled OR of 0.62 (95% CI: 0.28 to 1.36; *P* = 0.23) for the rapid meta-analysis. The relative difference was 6.89%, indicating that, in this case study, synthesizing evidence from RCTs registered on ClinicalTrials.gov is acceptable under a tolerance of 10%.

## Discussion

In this investigation, we explored the viability of utilizing the rapid evidence synthesis approach to expedite safety assessment via the ClinicalTrials.gov platform. Our analysis of the empirical dataset revealed that a median proportion of trials within a meta-analysis registered on ClinicalTrials.gov was 83.3%. Furthermore, we determined that rapid evidence synthesis, leveraging records from ClinicalTrials.gov, could be applied to 90% of meta-analyses focusing on adverse events. Notably, our empirical comparisons indicated that for safety outcomes, rapid evidence synthesis utilizing ClinicalTrials.gov yielded an acceptable effect estimate in 80% of meta-analyses under a 20% tolerable bias, along with accurate direction prediction in 90% of meta-analyses. These findings underscore the potential of leveraging ClinicalTrials.gov to accelerate the evidence synthesis process, providing valuable insights into safety evidence.

The accuracy of the rapid synthesis approach facilitated by ClinicalTrials.gov may have been underestimated in this study due to two conservative assumptions. Firstly, our identification of whether a trial had been registered relied on information provided in the original sources or published articles. This approach may have resulted in an undercounting of registered trials, as some trials could have been registered but failed to report this information in the articles [25]. Secondly, we assumed that trials for which we lacked access to the original sources or published articles were not registered on ClinicalTrials.gov. This assumption may have also led to an undercounting of registered trials, as some of these trials may have indeed been registered but remained unknown to us. Consequently, in real-world practice, the accuracy of the rapid synthesis approach is anticipated to be higher than indicated by the data presented in the current study.

We noticed that in the subgroup analysis, as the events rate goes down, both the point estimates and the significance of the *P*-value tend to be more consistent for the rapid approach and systematic approach. However, this is not necessarily a positive signal for safety assessment, at least for serious adverse events which had a very low incidence. The phenomenon suggested that the number of observed events was inadequate, so it is hard to distinguish the difference in the risk in terms of clinical perspective, and the power was low, so it is also impossible to distinguish the difference in the risk in terms of statistical inference. Unfortunately, there is currently no valid solution for this issue, except for awaiting more evidence from ongoing and planned trials.

It is important to highlight that a considerable portion of the meta-analyses within the reviews showed a notably low registration rate of RCTs on ClinicalTrials.gov. In fact, in a quarter of cases, the registration rate was 50% or less. Furthermore, a considerable proportion of registered RCTs, with a median of 14.3% (interquartile range: 0% to 33.3%), did not share their summarized data with the public. As the requirement for trial registration in a public registry, which was introduced in 2004 by the International Committee of Medical Journal Editors [26], trials initiated before 2004 were less likely to have been registered, and even for those initiated afterward, variations in registration practices exist among countries and specialties. The accuracy of rapid synthesis approaches is significantly influenced by the extent of trial registration and data sharing. The accuracy of rapid synthesis approaches is significantly impacted by the extent of trial registration and data sharing. Given the existence of other trial registries besides ClinicalTrials.gov, they could offer additional avenues for accessing trial results. It is anticipated that this situation will improve over time as the importance of registration becomes more widely recognized by researchers globally, and in many cases, is now mandated by funders.

To the best of our knowledge, this study represents the first exploration of employing the rapid evidence synthesis approach to expedite safety assessments using the ClinicalTrials.gov platform. Leveraging a large-scale real-world dataset ensures the results are both representative and conclusive. The insights obtained from this study hold particular relevance for policy-makers, guideline developers, evidence-producers, and evidence-users, offering valuable guidance for the more rapid utilization of evidence in decision-making processes. Furthermore, the feasibility demonstrated in conducting rapid reviews using data from ClinicalTrials.gov suggests the potential for automated evidence synthesis, given the structured nature of the data presented on the platform, which enhances readability and facilitates the extraction of pertinent information.

It is important to acknowledge several limitations inherent in our study. Firstly, our investigation primarily focused on safety outcomes in systematic reviews. However, safety issues may not immediately emerge in RCTs, as they are often relatively short-term, and some safety concerns may take longer to manifest. Therefore, RCT evidence may not provide a comprehensive picture of safety considerations. Additionally, our study exclusively concentrated on binary outcomes, and thus the findings may not be generalizable to other types of outcomes. Secondly, the systematic reviews included in our analysis were predominantly non-Cochrane reviews, which may not adhere to the same level of methodological rigor as Cochrane reviews, particularly in terms of the study identification process [27]. However, it is worth noting that even Cochrane reviews may not consistently adhere to the “gold standard” approach [28, 29]. Thirdly, as mentioned, due to the ambiguous definition of outcomes by some of the review authors, we were unable to address potential data extraction errors for relevant studies; consequently, such studies were excluded from the meta-analyses. Lastly, our study employed a relative recall method to identify trials. However, in real world application, individuals conducting rapid analyses may face challenges in finding trials on ClinicalTrials.gov using search terms similar to those used within the original systematic reviews. Moreover, the search interface of ClinicalTrials.gov may lack sophistication, potentially leading to less sensitive searches and the risk of overlooking relevant studies. Additionally, considering the multitude of other Trials registries and registry portals (such as ICTRP), exploring them to identify trial records could further enhance the value of this approach to rapid meta-analysis.

## Conclusions

Based on the current evidence, we conclude that accelerating evidence synthesisfor safety assessment, with a minimum of 5 RCTs, by using the ClinicalTrials.gov platform holds significant potential, as we can achieve acceptable prediction in a substantial proportion of meta-analyses. While at the same time, cautions are warranted that such a rapid procedure would lead to the information loss as researchers may not find all the relevant studies. To ensure the practical feasibility of this method, further crucial investigations should be undertaken, particularly focusing on developing search strategies on ClinicalTrials.gov and other registries to maximize the chances of identifying as many relevant studies as possible.

### Supplementary Information

**Supplementary Material 1.**

## Data Availability

Data could be obtained from the corresponding author upon request.
