# Supplementary material for: Accelerating evidence synthesis for safety assessment through ClinicalTrials.gov platform: a feasibility study
Source: BMC Med Res Methodol. 2024 Jul 30;24:165. doi: 10.1186/s12874-024-02225-2 (PMC11290241; doi:10.1186/s12874-024-02225-2)
Supplement: Supplementary file 1 — Supplementary Material 1. [file 12874_2024_2225_MOESM1_ESM.docx]

## Supplementary materials

## Accelerating evidence synthesis for safety assessment through ClinicalTrials.gov platform: A feasibility study

Tianqi Yu^1^, Xi Yang^2^, Justin Clark^3^, Lifeng Lin^4^, Luis Furuya-Kanamori^5^, Chang Xu^6^*

*^1^Center of Research in Epidemiology and Statistics, Université Paris Cité, Inserm, Paris, France*

*^2^**Department of Maternal, Child and Adolescent Health, School of Public Health,* *Anhui Medical University, Hefei, Anhui, China*

*^3^Centre for Research in Evidence-Based Practice (CREBP), Faculty of Health Sciences and Medicine, Bond University, Gold Coast, Queensland, Australia*

*^4^Department of Epidemiology and Biostatistics,* *University of* *Arizona, Tucson, AZ, USA*

*^5^School of Public Health, The University of Queensland, Herston, Australia*

*^6^Proof of Concept Center, Eastern Hepatobiliary Surgery Hospital, Third Affiliated Hospital, Second Military Medical University, Naval Medical University, Shanghai, China*

**Running title:** Accelerate evidence synthesis for safety assessment

**Correspondence to:** Chang Xu, Executive Director, Proof of Concept Center, Eastern Hepatobiliary Surgery Hospital, Third Affiliated Hospital, Second Military Medical University, Naval Medical University, Shanghai, China; Email: [xuchang2016@runbox.com](mailto:xuchang2016@runbox.com).

**Search Strategy (PubMed,** conducted on 28th-July, 2020**)**

1. "Systematic Reviews as Topic"[Mesh] OR "Systematic Review" [Publication Type] OR "Meta-Analysis as Topic"[Mesh] OR "Meta-Analysis" [Publication Type] OR "meta-analysis"[Title/Abstract] OR "meta analysis"[Title/Abstract] OR "systematic review"[Title/Abstract]
2. "randomized controlled trials as topic"[MeSH Major Topic] OR "clinical trials as topic"[MeSH Major Topic] OR "controlled clinical trials as topic"[MeSH Major Topic]
3. "randomized controlled trial*"[Title/Abstract] OR "controlled clinical trial*"[Title/Abstract] OR "clinical trial*"[Title/Abstract] OR "controlled trial*"[Title/Abstract] OR "trial*"[Title/Abstract]
4. "safety"[Title/Abstract] OR "harm*"[Title/Abstract] OR safe[Title/Abstract] OR poisoning[Title/Abstract] OR toxicity[Title/Abstract] OR tolerability[Title/Abstract] OR "complication*"[Title/Abstract] OR "adverse event*"[Title/Abstract] OR "adverse outcome*"[Title/Abstract] OR "untoward effect*"[Title/Abstract] OR "side effect*"[Title/Abstract] OR adverse n2 reaction[Title/Abstract]
5. #2 or #3
6. #1 AND #4 AND #5
7. Protocol[Title] OR overview [Title] OR "narrative review" [Title]
8. #6 NOT #7
9. (#8) AND (("2018/01/02"[Date - Publication]: "2020/01/01"[Date - Publication])) AND (humans[Filter]) Filters: Humans
10. (#8) AND (("2015/01/01"[Date - Publication]: "2018/01/01"[Date - Publication])) AND (humans[Filter]) Filters: Humans
11. #9 or # 10

**Table S1. Example data — suicidal ideation events associated with varenicline.**

| **Study** | **Intervention** | | **Control** | | **Registered**  **(Y/N)** | **Platforms** | **NCT Number** | **Posted Results** |
| --- | --- | --- | --- | --- | --- | --- | --- | --- |
|  | **Events** | **Total** | **Events** | **Total** |  |  |  |  |
| Jorenby 2006 | 0 | 343 | 1 | 340 | Y | NCT | NCT00143364 | N |
| Hughes 2011 | 0 | 107 | 1 | 111 | N | NA | NA | NA |
| Zhao 2011 | 0 | 14 | 0 | 10 | N | NA | NA | NA |
| Brandon 2011 | 0 | 46 | 0 | 54 | N | NA | NA | NA |
| Ebbert 2011 | 0 | 38 | 0 | 38 | N | NA | NA | NA |
| Steinberg 2011 | 0 | 40 | 0 | 39 | N | NA | NA | NA |
| Bolliger 2011 | 2 | 390 | 0 | 198 | Y | NCT | NCT00594204 | Y |
| Tashkin 2011 | 0 | 248 | 1 | 251 | Y | NCT | NCT00285012 | Y |
| Williams 2012 | 5 | 84 | 3 | 43 | Y | NCT | NCT00644969 | Y |
| Rennard 2012 | 1 | 486 | 2 | 165 | Y | NCT | NCT00691483 | Y |
| Mitchell 2012 | 0 | 33 | 1 | 31 | Y | NCT | NCT01011907 | Y |
| Wong 2012 | 0 | 151 | 0 | 135 | Y | NCT | NCT01320462 | N |
| McClure 2013 | 0 | 41 | 0 | 42 | N | NA | NA | NA |
| Stein 2013 | 1 | 111 | 0 | 33 | Y | NCT | NCT00790569 | Y |
| Cinciripini 2013 | 0 | 86 | 1 | 106 | Y | NCT | NCT00507728 | Y |
| Meszaros 2013 | 2 | 5 | 2 | 5 | Y | NCT | NCT00727103 | N |
| Anthenelli 2013 | 0 | 256 | 3 | 269 | Y | NCT | NCT01078298 | Y |
| Evins 2014 | 2 | 40 | 2 | 47 | Y | NCT | NCT00621777 | Y |
| Chengappa 2014 | 2 | 31 | 1 | 29 | Y | NCT | NCT01010204 | Y |
| Gonzales 2014 | 0 | 249 | 0 | 245 | Y | NCT | NCT01244061 | Y |

**List of 151 eligible systematic reviews:**

1. Abdel-Qadir H, Ethier JL, Lee DS, Thavendiranathan P, Amir E. Cardiovascular toxicity of angiogenesis inhibitors in treatment of malignancy: A systematic review and meta-analysis. Cancer Treat Rev. 2017;53:120-127. doi:10.1016/j.ctrv.2016.12.002
2. Abdel-Rahman O, ElHalawani H, Essam-Eldin S. S-1-based regimens and the risk of leucopenic complications; a Meta-analysis with comparison to other fluoropyrimidines and non fluoropyrimidines. Expert Opin Drug Saf. 2016;15(4):437-448. doi:10.1517/14740338.2016.1146674
3. Abdel-Rahman O, ElHalawani H, Fouad M. Risk of endocrine complications in cancer patients treated with immune check point inhibitors: a meta-analysis. Future Oncol. 2016;12(3):413-425. doi:10.2217/fon.15.222
4. Abdel-Rahman O, ElHalawani H, Fouad M. Risk of gastrointestinal complications in cancer patients treated with immune checkpoint inhibitors: a meta-analysis. Immunotherapy. 2015;7(11):1213-1227. doi:10.2217/imt.15.87
5. Abdel-Rahman O, ElHalawani H. Risk of cardiovascular adverse events in patients with solid tumors treated with ramucirumab: A meta analysis and summary of other VEGF targeted agents. Crit Rev Oncol Hematol. 2016;102:89-100. doi:10.1016/j.critrevonc.2016.04.003
6. Abdel-Rahman O, ElHalawani H. Risk of hematological toxicities in patients with solid tumors treated with ramucirumab: a meta-analysis. Future Oncol. 2015;11(21):2949-2961. doi:10.2217/fon.15.178
7. Abdel-Rahman O, Fouad M. Risk of mucocutaneous toxicities in patients with solid tumors treated with lapatinib: a systematic review and meta-analysis. Curr Med Res Opin. 2015;31(5):975-986. doi:10.1185/03007995.2015.1020367
8. Abdel-Rahman O, Fouad M. Risk of oral and gastrointestinal mucosal injury in patients with solid tumors treated with everolimus, temsirolimus or ridaforolimus: a comparative systematic review and meta-analysis. Expert Rev Anticancer Ther. 2015;15(7):847-858. doi:10.1586/14737140.2015.1047350
9. Abdel-Rahman O, Helbling D, Schmidt J, et al. Treatment-associated Fatigue in Cancer Patients Treated with Immune Checkpoint Inhibitors; a Systematic Review and Meta-analysis. Clin Oncol (R Coll Radiol). 2016;28(10):e127-e138.
10. Ammann EM, Haskins CB, Fillman KM, et al. Intravenous immune globulin and thromboembolic adverse events: A systematic review and meta-analysis of RCTs. Am J Hematol. 2016;91(6):594-605. doi:10.1002/ajh.24358
11. Baxi S, Yang A, Gennarelli RL, et al. Immune-related adverse events for anti-PD-1 and anti-PD-L1 drugs: systematic review and meta-analysis. BMJ. 2018;360:k793.
12. Bilal J, Berlinberg A, Riaz IB, et al. Risk of Infections and Cancer in Patients With Rheumatologic Diseases Receiving Interleukin Inhibitors: A Systematic Review and Meta-analysis. JAMA Netw Open. 2019;2(10):e1913102. Published 2019 Oct 2. doi:10.1001/jamanetworkopen.2019.13102
13. Boltman-Binkowski H. A systematic review: Are herbal and homeopathic remedies used during pregnancy safe?. Curationis. 2016;39(1):1514. Published 2016 Apr 13. doi:10.4102/curationis.v39i1.1514
14. Bolton M, Hodkinson A, Boda S, et al. Serious adverse events reported in placebo randomised controlled trials of oral naltrexone: a systematic review and meta-analysis. BMC Med. 2019;17(1):10. Published 2019 Jan 15. doi:10.1186/s12916-018-1242-0
15. Bonovas S, Minozzi S, Lytras T, et al. Risk of malignancies using anti-TNF agents in rheumatoid arthritis, psoriatic arthritis, and ankylosing spondylitis: a systematic review and meta-analysis. Expert Opin Drug Saf. 2016;15(sup1):35-54. doi:10.1080/14740338.2016.1238458
16. Botero Aguirre JP, Restrepo Hamid AM. Amphotericin B deoxycholate versus liposomal amphotericin B: effects on kidney function. Cochrane Database Syst Rev. 2015;(11):CD010481.
17. Bundhun PK, Janoo G, Huang F. Adverse drug events observed in patients with type 2 diabetes mellitus treated with 100 mg versus 300 mg canagliflozin: a systematic review and meta-analysis of published randomized controlled trials. BMC Pharmacol Toxicol. 2017;18(1):19. Published 2017 Apr 16. doi:10.1186/s40360-017-0126-9
18. Cai Q, Feng L, Yap KZ. Systematic review and meta-analysis of reported adverse events of long-term intranasal oxytocin treatment for autism spectrum disorder. Psychiatry Clin Neurosci. 2018;72(3):140-151. doi:10.1111/pcn.12627
19. Caldeira D, Alves D, Costa J, Ferreira JJ, Pinto FJ. Ibrutinib increases the risk of hypertension and atrial fibrillation: Systematic review and meta-analysis. PLoS One. 2019;14(2):e0211228. Published 2019 Feb 20. doi:10.1371/journal.pone.0211228
20. Caldeira D, Barra M, Ferreira A, et al. Systematic review with meta-analysis: the risk of major gastrointestinal bleeding with non-vitamin K antagonist oral anticoagulants. Aliment Pharmacol Ther. 2015;42(11-12):1239-1249. doi:10.1111/apt.13412
21. Caldeira D, Barra M, Santos AT, de Abreu D, Costa J, Ferreira JJ. Risk of insomnia with non-vitamin K oral anticoagulants: systematic review and meta-analysis. Sleep Breath. 2015;19(3):1043-1049. doi:10.1007/s11325-014-1112-8
22. Caldeira D, Canastro M, Barra M, et al. Risk of Substantial Intraocular Bleeding With Novel Oral Anticoagulants: Systematic Review and Meta-analysis. JAMA Ophthalmol. 2015;133(7):834-839. doi:10.1001/jamaophthalmol.2015.0985
23. Caldeira D, Rodrigues FB, Barra M, et al. Non-vitamin K antagonist oral anticoagulants and major bleeding-related fatality in patients with atrial fibrillation and venous thromboembolism: a systematic review and meta-analysis. Heart. 2015;101(15):1204-1211. doi:10.1136/heartjnl-2015-307489
24. Caldeira D, Rodrigues FB, Duarte MM, et al. Cardiac Harms of Sofosbuvir: Systematic Review and Meta-Analysis. Drug Saf. 2018;41(1):77-86. doi:10.1007/s40264-017-0586-2
25. Capogrosso Sansone A, Mantarro S, Tuccori M, et al. Safety Profile of Certolizumab Pegol in Patients with Immune-Mediated Inflammatory Diseases: A Systematic Review and Meta-Analysis. Drug Saf. 2015;38(10):869-888.
26. Catalá-López F, Corrales I, de la Fuente-Honrubia C, et al. Risk of thromboembolism with thrombopoietin receptor agonists in adult patients with thrombocytopenia: Systematic review and meta-analysis of randomized controlled trials. Med Clin (Barc). 2015;145(12):511-519. doi:10.1016/j.medcli.2015.03.014
27. Cates CJ, Schmidt S, Ferrer M, Sayer B, Waterson S. Inhaled steroids with and without regular salmeterol for asthma: serious adverse events. Cochrane Database Syst Rev. 2018;12(12):CD006922.
28. Chen F, Pu F. Safety of Denosumab Versus Zoledronic Acid in Patients with Bone Metastases: A Meta-Analysis of Randomized Controlled Trials. Oncol Res Treat. 2016;39(7-8):453-459. doi:10.1159/000447372
29. Chen J, Lu Y, Zheng Y. Incidence and risk of hypertension with bevacizumab in non-small-cell lung cancer patients: a meta-analysis of randomized controlled trials. Drug Des Devel Ther. 2015;9:4751-4760. Published 2015 Aug 18. doi:10.2147/DDDT.S87258
30. Ciccarese C, Iacovelli R, Bria E, et al. The incidence and relative risk of pulmonary toxicity in patients treated with anti-PD1/PD-L1 therapy for solid tumors: a meta-analysis of current studies. Immunotherapy. 2017;9(7):579-587.
31. Conway R, Low C, Coughlan RJ, O'Donnell MJ, Carey JJ. Leflunomide Use and Risk of Lung Disease in Rheumatoid Arthritis: A Systematic Literature Review and Metaanalysis of Randomized Controlled Trials. J Rheumatol. 2016;43(5):855-860.
32. Conway R, Low C, Coughlan RJ, O'Donnell MJ, Carey JJ. Methotrexate use and risk of lung disease in psoriasis, psoriatic arthritis, and inflammatory bowel disease: systematic literature review and meta-analysis of randomised controlled trials. BMJ. 2015;350:h1269.
33. Conway R, Low C, Coughlan RJ, O'Donnell MJ, Carey JJ. Risk of liver injury among methotrexate users: A meta-analysis of randomised controlled trials. Semin Arthritis Rheum. 2015;45(2):156-162.
34. Cui R, Chu L, Liu ZQ, et al. Hematologic toxicity assessment in solid tumor patients treated with cetuximab: a pooled analysis of 18 randomized controlled trials [published correction appears in Int J Cancer. 2016 Apr 15;138(8):E4]. Int J Cancer. 2015;136(4):936-944.
35. Dahal A, Bellows BK, Sonpavde G, et al. Incidence of Severe Nephrotoxicity With Cisplatin Based on Renal Function Eligibility Criteria: Indirect Comparison Meta-analysis. Am J Clin Oncol. 2016;39(5):497-506.
36. Dahal K, Sharma S, Yousuf A, et al. A comparison of standard versus low dose heparin on access-related complications after coronary angiography through radial access: A meta-analysis of randomized controlled trials. Cardiovasc Revasc Med. 2018;19(5 Pt B):575-579. doi:10.1016/j.carrev.2017.10.018
37. Dicembrini I, Tomberli B, Nreu B, et al. Peripheral artery disease and amputations with Sodium-Glucose co-Transporter-2 (SGLT-2) inhibitors: A meta-analysis of randomized controlled trials. Diabetes Res Clin Pract. 2019;153:138-144. doi:10.1016/j.diabres.2019.05.028
38. Donnan JR, Grandy CA, Chibrikov E, et al. Comparative safety of the sodium glucose co-transporter 2 (SGLT2) inhibitors: a systematic review and meta-analysis. BMJ Open. 2019;9(1):e022577. Published 2019 Feb 1. doi:10.1136/bmjopen-2018-022577
39. Dore MP, Bibbò S, Fresi G, Bassotti G, Pes GM. Side Effects Associated with Probiotic Use in Adult Patients with Inflammatory Bowel Disease: A Systematic Review and Meta-Analysis of Randomized Controlled Trials. Nutrients. 2019;11(12):2913. Published 2019 Dec 2. doi:10.3390/nu11122913
40. Elgendy IY, Mahmoud AN, Barakat AF, et al. Cardiovascular Safety of Dipeptidyl-Peptidase IV Inhibitors: A Meta-Analysis of Placebo-Controlled Randomized Trials. Am J Cardiovasc Drugs. 2017;17(2):143-155. doi:10.1007/s40256-016-0208-x
41. Farah D, Leme GM, Eliaschewitz FG, Fonseca MCM. A safety and tolerability profile comparison between dipeptidyl peptidase-4 inhibitors and sulfonylureas in diabetic patients: A systematic review and meta-analysis. Diabetes Res Clin Pract. 2019;149:47-63. doi:10.1016/j.diabres.2019.01.025
42. Feng X, Tian M, Zhang W, Mei H. Gastrointestinal safety of etoricoxib in osteoarthritis and rheumatoid arthritis: A meta-analysis. PLoS One. 2018;13(1):e0190798. Published 2018 Jan 10. doi:10.1371/journal.pone.0190798
43. Funakoshi T, Suzuki M, Muss HB. Infection risk in breast cancer patients treated with trastuzumab: a systematic review and meta-analysis. Breast Cancer Res Treat. 2015;149(2):321-330. doi:10.1007/s10549-014-3184-3
44. Gafter-Gvili A, Gurion R, Raanani P, Shpilberg O, Vidal L. Bendamustine-associated infections-systematic review and meta-analysis of randomized controlled trials. Hematol Oncol. 2017;35(4):424-431.
45. Geng Z, Yu Y, Hu S, Dong L, Ye C. Tocilizumab and the risk of respiratory adverse events in patients with rheumatoid arthritis: a systematic review and meta-analysis of randomised controlled trials. Clin Exp Rheumatol. 2019;37(2):318-323.
46. Ghatalia P, Je Y, Nguyen PL, Trinh QD, Choueiri TK, Sonpavde G. Fatigue with vascular endothelial growth factor receptor tyrosine kinase inhibitors and mammalian target of rapamycin inhibitors in patients with renal cell carcinoma (RCC) and other malignancies: A meta-analysis of randomized clinical trials. Crit Rev Oncol Hematol. 2015;95(2):251-263. doi:10.1016/j.critrevonc.2015.03.006
47. Gillies M, Ranakusuma A, Hoffmann T, et al. Common harms from amoxicillin: a systematic review and meta-analysis of randomized placebo-controlled trials for any indication. CMAJ. 2015;187(1):E21-E31. doi:10.1503/cmaj.140848
48. Gu B, Gao W, Chu H, et al. Adverse events risk associated with anti-VEGFR agents in the treatment of advanced nonsmall-cell lung cancer: A meta-analysis. Medicine (Baltimore). 2016;95(48):e3752.
49. Guo X, Yang Q, Dong J, Liao L, Zhang W, Liu F. Tumour Risk with Once-Weekly Glucagon-Like Peptide-1 Receptor Agonists in Type 2 Diabetes Mellitus Patients: A Systematic Review. Clin Drug Investig. 2016;36(6):433-441. doi:10.1007/s40261-016-0389-8
50. Gyawali B, Shimokata T, Ando M, Honda K, Ando Y. Risk of serious adverse events and fatal adverse events with sorafenib in patients with solid cancer: a meta-analysis of phase 3 randomized controlled trials†. Ann Oncol. 2017;28(2):246-253.
51. Hansen MP, Scott AM, McCullough A, et al. Adverse events in people taking macrolide antibiotics versus placebo for any indication. Cochrane Database Syst Rev. 2019;1(1):CD011825. Published 2019 Jan 18. doi:10.1002/14651858.CD011825.pub2
52. Hao S, Tian W, Gao B, et al. Does dual HER-2 blockade treatment increase the risk of severe toxicities of special interests in breast cancer patients: A meta-analysis of randomized controlled trials. Oncotarget. 2017;8(12):19923-19933. doi:10.18632/oncotarget.15252
53. Hill AM, Mitchell N, Hughes S, Pozniak AL. Risks of cardiovascular or central nervous system adverse events and immune reconstitution inflammatory syndrome, for dolutegravir versus other antiretrovirals: meta-analysis of randomized trials. Curr Opin HIV AIDS. 2018;13(2):102-111.
54. Holmskov M, Storebø OJ, Moreira-Maia CR, et al. Gastrointestinal adverse events during methylphenidate treatment of children and adolescents with attention deficit hyperactivity disorder: A systematic review with meta-analysis and Trial Sequential Analysis of randomised clinical trials. PLoS One. 2017;12(6):e0178187. Published 2017 Jun 15. doi:10.1371/journal.pone.0178187
55. Hong D, Zhang G, Zhang X, Lian X. Pulmonary Toxicities of Gefitinib in Patients With Advanced Non-Small-Cell Lung Cancer: A Meta-Analysis of Randomized Controlled Trials [published correction appears in Medicine (Baltimore). 2016 Jul 29;95(30):e156a]. Medicine (Baltimore). 2016;95(9):e3008.
56. Honvo G, Leclercq V, Geerinck A, et al. Safety of Topical Non-steroidal Anti-Inflammatory Drugs in Osteoarthritis: Outcomes of a Systematic Review and Meta-Analysis. Drugs Aging. 2019;36(Suppl 1):45-64. doi:10.1007/s40266-019-00661-0
57. Honvo G, Reginster JY, Rabenda V, et al. Safety of Symptomatic Slow-Acting Drugs for Osteoarthritis: Outcomes of a Systematic Review and Meta-Analysis. Drugs Aging. 2019;36(Suppl 1):65-99. doi:10.1007/s40266-019-00662-z
58. Honvo G, Reginster JY, Rannou F, et al. Safety of Intra-articular Hyaluronic Acid Injections in Osteoarthritis: Outcomes of a Systematic Review and Meta-Analysis. Drugs Aging. 2019;36(Suppl 1):101-127. doi:10.1007/s40266-019-00657-w
59. Hu Y, Xu W, Cao F. A meta-analysis of randomized controlled trials: combination of ketamine and propofol versus ketamine alone for procedural sedation and analgesia in children. Intern Emerg Med. 2019;14(7):1159-1165. doi:10.1007/s11739-019-02173-6
60. Hua Q, Zhu Y, Liu H. Severe and fatal adverse events risk associated with rituximab addition to B-cell non-Hodgkin's lymphoma (B-NHL) chemotherapy: a meta-analysis. J Chemother. 2015;27(6):365-370. doi:10.1179/1973947815Y.0000000025
61. Huang F, Luo ZC. Adverse drug events associated with 5mg versus 10mg Tofacitinib (Janus kinase inhibitor) twice daily for the treatment of autoimmune diseases: A systematic review and meta-analysis of randomized controlled trials. Clin Rheumatol. 2019;38(2):523-534. doi:10.1007/s10067-018-4299-4
62. Huang ST, Tian BS, Xiao O, Yang YJ, Zhou SY. Safety of antivascular endothelial growth factor administration in the ocular anterior segment in pterygium and neovascular glaucoma treatment: Systematic review and meta-analysis. Medicine (Baltimore). 2018;97(34):e11960. doi:10.1097/MD.0000000000011960
63. Jalili M, Bahreini M, Doosti-Irani A, Masoomi R, Arbab M, Mirfazaelian H. Ketamine-propofol combination (ketofol) vs propofol for procedural sedation and analgesia: systematic review and meta-analysis. Am J Emerg Med. 2016;34(3):558-569. doi:10.1016/j.ajem.2015.12.074
64. Janjua S, Schmidt S, Ferrer M, Cates CJ. Inhaled steroids with and without regular formoterol for asthma: serious adverse events. Cochrane Database Syst Rev. 2019;9(9):CD006924. Published 2019 Sep 25. doi:10.1002/14651858.CD006924.pub4
65. Jia Z, Lu H, Yang X, et al. Adverse Events of Botulinum Toxin Type A in Facial Rejuvenation: A Systematic Review and Meta-Analysis. Aesthetic Plast Surg. 2016;40(5):769-777. doi:10.1007/s00266-016-0682-1
66. Johansen KGV, Tarp S, Astrup A, Lund H, Pagsberg AK, Christensen R. Harms associated with taking nalmefene for substance use and impulse control disorders: A systematic review and meta-analysis of randomised controlled trials. PLoS One. 2017;12(8):e0183821. Published 2017 Aug 29. doi:10.1371/journal.pone.0183821
67. Khan AR, Bavishi C, Riaz H, et al. Increased Risk of Adverse Neurocognitive Outcomes With Proprotein Convertase Subtilisin-Kexin Type 9 Inhibitors. Circ Cardiovasc Qual Outcomes. 2017;10(1):e003153.
68. Khosrow-Khavar F, Filion KB, Al-Qurashi S, et al. Cardiotoxicity of aromatase inhibitors and tamoxifen in postmenopausal women with breast cancer: a systematic review and meta-analysis of randomized controlled trials. Ann Oncol. 2017;28(3):487-496. doi:10.1093/annonc/mdw673
69. Kim DH, Rogers JR, Fulchino LA, Kim CA, Solomon DH, Kim SC. Bisphosphonates and risk of cardiovascular events: a meta-analysis. PLoS One. 2015;10(4):e0122646.
70. Lao KS, He Y, Wong IC, Besag FM, Chan EW. Tolerability and Safety Profile of Cariprazine in Treating Psychotic Disorders, Bipolar Disorder and Major Depressive Disorder: A Systematic Review with Meta-Analysis of Randomized Controlled Trials. CNS Drugs. 2016;30(11):1043-1054. doi:10.1007/s40263-016-0382-z
71. Lew S, Chamberlain RS. Risk of Metabolic Complications in Patients with Solid Tumors Treated with mTOR inhibitors: Meta-analysis. Anticancer Res. 2016;36(4):1711-1718.
72. Li H, Wang C, Zhang S, et al. Safety Profile of Atorvastatin 80 mg: A Meta-Analysis of 17 Randomized Controlled Trials in 21,910 Participants. Drug Saf. 2016;39(5):409-419. doi:10.1007/s40264-016-0394-0
73. Li J, Gu J. Cardiovascular Toxicities with Vascular Endothelial Growth Factor Receptor Tyrosine Kinase Inhibitors in Cancer Patients: A Meta-Analysis of 77 Randomized Controlled Trials. Clin Drug Investig. 2018;38(12):1109-1123. doi:10.1007/s40261-018-0709-2
74. Li J, Gu J. Rash and Pruritus With PD-1 Inhibitors in Cancer Patients: A Meta-Analysis of Randomized Controlled Trials. J Clin Pharmacol. 2019;59(1):45-54. doi:10.1002/jcph.1291
75. Li J, Yan H. Skin toxicity with anti-EGFR monoclonal antibody in cancer patients: a meta-analysis of 65 randomized controlled trials. Cancer Chemother Pharmacol. 2018;82(4):571-583. doi:10.1007/s00280-018-3644-2
76. Li X, Wan J, Wu Z, et al. Fatal adverse events with molecular targeted agents in the treatment of advanced hepatocellular carcinoma: a meta-analysis of randomized controlled trials. Drug Des Devel Ther. 2018;12:3043-3049. Published 2018 Sep 18. doi:10.2147/DDDT.S151241
77. Liang XJ, Shen J. Adverse events risk associated with angiogenesis inhibitors addition to therapy in ovarian cancer: a meta-analysis of randomized controlled trials. Eur Rev Med Pharmacol Sci. 2016;20(12):2701-2709.
78. Liu B, Ding F, Liu Y, et al. Incidence and risk of hypertension associated with vascular endothelial growth factor receptor tyrosine kinase inhibitors in cancer patients: a comprehensive network meta-analysis of 72 randomized controlled trials involving 30013 patients. Oncotarget. 2016;7(41):67661-67673. doi:10.18632/oncotarget.11813
79. Liu W, Ma X, Zhou W. Adverse events of benralizumab in moderate to severe eosinophilic asthma: A meta-analysis. Medicine (Baltimore). 2019;98(22):e15868. doi:10.1097/MD.0000000000015868
80. Liu Y, Qi M, Hou S, et al. Risk of rash associated with vandetanib treatment in non-small-cell lung cancer patients: A meta-analysis of 9 randomized controlled trials. Medicine (Baltimore). 2017;96(43):e8345. doi:10.1097/MD.0000000000008345
81. Liu Y, Zhang X, Chai S, Zhao X, Ji L. Risk of Malignant Neoplasia with Glucagon-Like Peptide-1 Receptor Agonist Treatment in Patients with Type 2 Diabetes: A Meta-Analysis. J Diabetes Res. 2019;2019:1534365. Published 2019 Jul 16. doi:10.1155/2019/1534365
82. Luo L, Yuan X, Huang W, et al. Safety of coadministration of ezetimibe and statins in patients with hypercholesterolaemia: a meta-analysis. Intern Med J. 2015;45(5):546-557. doi:10.1111/imj.12706
83. Luo W, Wang Z, Tian P, Li W. Safety and tolerability of PD-1/PD-L1 inhibitors in the treatment of non-small cell lung cancer: a meta-analysis of randomized controlled trials. J Cancer Res Clin Oncol. 2018;144(10):1851-1859. doi:10.1007/s00432-018-2707-4
84. Lv WW, Zhang JJ, Zhou XL, Song Z, Wei CM. Safety of combining vascular endothelial growth factor receptor tyrosine-kinase inhibitors with chemotherapy in patients with advanced non-small-cell lung cancer: A PRISMA-compliant meta-analysis. Medicine (Baltimore). 2019;98(23):e15806. doi:10.1097/MD.0000000000015806
85. Ma C, Panaccione NR, Nguyen TM, et al. Adverse Events and Nocebo Effects in Inflammatory Bowel Disease: A Systematic Review and Meta-Analysis of Randomized Controlled Trials. J Crohns Colitis. 2019;13(9):1201-1216.
86. Ma H, Liu Y, Huang L, et al. The Adverse Events of Oxycodone in Cancer-Related Pain: A Systematic Review and Meta-Analysis of Randomized Controlled Trials. Medicine (Baltimore). 2016;95(15):e3341. doi:10.1097/MD.0000000000003341
87. Man J, Ritchie G, Links M, Lord S, Lee CK. Treatment-related toxicities of immune checkpoint inhibitors in advanced cancers: A meta-analysis. Asia Pac J Clin Oncol. 2018;14(3):141-152. doi:10.1111/ajco.12838
88. Martel S, Bruzzone M, Ceppi M, et al. Risk of adverse events with the addition of targeted agents to endocrine therapy in patients with hormone receptor-positive metastatic breast cancer: A systematic review and meta-analysis. Cancer Treat Rev. 2018;62:123-132. doi:10.1016/j.ctrv.2017.09.009
89. Mincu RI, Mahabadi AA, Michel L, et al. Cardiovascular Adverse Events Associated With BRAF and MEK Inhibitors: A Systematic Review and Meta-analysis. JAMA Netw Open. 2019;2(8):e198890. Published 2019 Aug 2. doi:10.1001/jamanetworkopen.2019.8890
90. Minozzi S, Bonovas S, Lytras T, et al. Risk of infections using anti-TNF agents in rheumatoid arthritis, psoriatic arthritis, and ankylosing spondylitis: a systematic review and meta-analysis. Expert Opin Drug Saf. 2016;15(sup1):11-34. doi:10.1080/14740338.2016.1240783
91. Miroddi M, Sterrantino C, Simmonds M, et al. Systematic review and meta-analysis of the risk of severe and life-threatening thromboembolism in cancer patients receiving anti-EGFR monoclonal antibodies (cetuximab or panitumumab). Int J Cancer. 2016;139(10):2370-2380. doi:10.1002/ijc.30280
92. Miroddi M, Sterrantino C, Simonelli I, Ciminata G, Phillips RS, Calapai G. Risk of grade 3-4 diarrhea and mucositis in colorectal cancer patients receiving anti-EGFR monoclonal antibodies regimens: A meta-analysis of 18 randomized controlled clinical trials. Crit Rev Oncol Hematol. 2015;96(2):355-371. doi:10.1016/j.critrevonc.2015.06.004
93. Misawa F, Kishimoto T, Hagi K, Kane JM, Correll CU. Safety and tolerability of long-acting injectable versus oral antipsychotics: A meta-analysis of randomized controlled studies comparing the same antipsychotics. Schizophr Res. 2016;176(2-3):220-230. doi:10.1016/j.schres.2016.07.018
94. pedro Luiz Spinelli CoelhoF, Luo ZC. Risk of Adverse Drug Events Observed with Baricitinib 2 mg Versus Baricitinib 4 mg Once Daily for the Treatment of Rheumatoid Arthritis: A Systematic Review and Meta-Analysis of Randomized Controlled Trials. BioDrugs. 2018;32(5):415-423. doi:10.1007/s40259-018-0304-3
95. Penninga EI, Graudal N, Ladekarl MB, Jürgens G. Adverse Events Associated with Flumazenil Treatment for the Management of Suspected Benzodiazepine Intoxication--A Systematic Review with Meta-Analyses of Randomised Trials. Basic Clin Pharmacol Toxicol. 2016;118(1):37-44. doi:10.1111/bcpt.12434
96. Pozzi M, Carnovale C, Peeters GGAM, et al. Adverse drug events related to mood and emotion in paediatric patients treated for ADHD: A meta-analysis. J Affect Disord. 2018;238:161-178. doi:10.1016/j.jad.2018.05.021
97. Puckrin R, Saltiel MP, Reynier P, Azoulay L, Yu OHY, Filion KB. SGLT-2 inhibitors and the risk of infections: a systematic review and meta-analysis of randomized controlled trials. Acta Diabetol. 2018;55(5):503-514. doi:10.1007/s00592-018-1116-0
98. Qi WX, Fu S, Zhang Q, Guo XM. Bevacizumab increases the risk of infections in cancer patients: A systematic review and pooled analysis of 41 randomized controlled trials. Crit Rev Oncol Hematol. 2015;94(3):323-336. doi:10.1016/j.critrevonc.2015.02.007
99. Quagliato LA, Cosci F, Shader RI, et al. Selective serotonin reuptake inhibitors and benzodiazepines in panic disorder: A meta-analysis of common side effects in acute treatment. J Psychopharmacol. 2019;33(11):1340-1351. doi:10.1177/0269881119859372
100. Raccah BH, Perlman A, Danenberg HD, Pollak A, Muszkat M, Matok I. Major Bleeding and Hemorrhagic Stroke With Direct Oral Anticoagulants in Patients With Renal Failure: Systematic Review and Meta-Analysis of Randomized Trials. Chest. 2016;149(6):1516-1524.
101. Rogers SC, Garcia CA, Wu S. Discontinuation of Everolimus Due to Related and Unrelated Adverse Events in Cancer Patients: A Meta-Analysis. Cancer Invest. 2017;35(8):552-561. doi:10.1080/07357907.2017.1344697
102. Roviello G, Generali D. Is the fatigue an adverse event of the second generation of hormonal therapy? Data from a literature-based meta-analysis. Med Oncol. 2018;35(3):29. Published 2018 Jan 31. doi:10.1007/s12032-018-1081-z
103. Rungapiromnan W, Yiu ZZN, Warren RB, Griffiths CEM, Ashcroft DM. Impact of biologic therapies on risk of major adverse cardiovascular events in patients with psoriasis: systematic review and meta-analysis of randomized controlled trials. Br J Dermatol. 2017;176(4):890-901. doi:10.1111/bjd.14964
104. Santoni M, Guerra F, Conti A, et al. Incidence and risk of cardiotoxicity in cancer patients treated with targeted therapies. Cancer Treat Rev. 2017;59:123-131. doi:10.1016/j.ctrv.2017.07.006
105. Sardar P, Udell JA, Chatterjee S, Bansilal S, Mukherjee D, Farkouh ME. Effect of Intensive Versus Standard Blood Glucose Control in Patients With Type 2 Diabetes Mellitus in Different Regions of the World: Systematic Review and Meta-analysis of Randomized Controlled Trials. J Am Heart Assoc. 2015;4(5):e001577. Published 2015 May 5. doi:10.1161/JAHA.114.001577
106. Shah ED, Farida JP, Siegel CA, Chong K, Melmed GY. Risk for Overall Infection with Anti-TNF and Anti-integrin Agents Used in IBD: A Systematic Review and Meta-analysis. Inflamm Bowel Dis. 2017;23(4):570-577. doi:10.1097/MIB.0000000000001049
107. Sterling LH, Windle SB, Filion KB, Touma L, Eisenberg MJ. Varenicline and Adverse Cardiovascular Events: A Systematic Review and Meta-Analysis of Randomized Controlled Trials. J Am Heart Assoc. 2016;5(2):e002849. Published 2016 Feb 22. doi:10.1161/JAHA.115.002849
108. Su Q, Zhang XC, Wang DY, et al. The risk of immune-related endocrine disorders associated with anti-PD-1 inhibitors therapy for solid tumors: A systematic review and meta-analysis. Int Immunopharmacol. 2018;59:328-338. doi:10.1016/j.intimp.2018.04.021
109. Sun W, Li J. Skin Toxicities with Epidermal Growth Factor Receptor Tyrosine Kinase Inhibitors in Cancer Patients: A Meta-Analysis of Randomized Controlled Trials. Cancer Invest. 2019;37(6):253-264. doi:10.1080/07357907.2019.1634089
110. Tandan M, Cormican M, Vellinga A. Adverse events of fluoroquinolones vs. other antimicrobials prescribed in primary care: A systematic review and meta-analysis of randomized controlled trials. Int J Antimicrob Agents. 2018;52(5):529-540. doi:10.1016/j.ijantimicag.2018.04.014
111. Tang B, Wang J, Luo LL, Li QG, Huang D. Risks of budesonide/formoterol for the treatment of stable COPD: a meta-analysis. Int J Chron Obstruct Pulmon Dis. 2019;14:757-766. Published 2019 Apr 1. doi:10.2147/COPD.S192166
112. Thomas KH, Martin RM, Knipe DW, Higgins JP, Gunnell D. Risk of neuropsychiatric adverse events associated with varenicline: systematic review and meta-analysis. BMJ. 2015;350:h1109. Published 2015 Mar 12. doi:10.1136/bmj.h1109
113. Thomopoulos C, Parati G, Zanchetti A. Effects of blood-pressure-lowering treatment in hypertension: 9. Discontinuations for adverse events attributed to different classes of antihypertensive drugs: meta-analyses of randomized trials. J Hypertens. 2016;34(10):1921-1932.
114. Tong H, Zhu Y, Liu Y. Incidence and risk of fatigue in cancer patients treated with MET inhibitors: A systematic review and meta-analysis. Medicine (Baltimore). 2019;98(22):e15522. doi:10.1097/MD.0000000000015522
115. Tong S, Fan K, Jiang K, et al. Increased risk of severe infections in non-small-cell lung cancer patients treated with pemetrexed: a meta-analysis of randomized controlled trials. Curr Med Res Opin. 2017;33(1):31-37. doi:10.1080/03007995.2016.1232705
116. Totzeck M, Mincu RI, Rassaf T. Cardiovascular Adverse Events in Patients With Cancer Treated With Bevacizumab: A Meta-Analysis of More Than 20 000 Patients. J Am Heart Assoc. 2017;6(8):e006278. Published 2017 Aug 10. doi:10.1161/JAHA.117.006278
117. Varma A, Zis P. Nocebo effect in myasthenia gravis: systematic review and meta-analysis of placebo-controlled clinical trials. Acta Neurol Belg. 2019;119(2):257-264.
118. Varvaki Rados D, Catani Pinto L, Reck Remonti L, Bauermann Leitão C, Gross JL. The Association between Sulfonylurea Use and All-Cause and Cardiovascular Mortality: A Meta-Analysis with Trial Sequential Analysis of Randomized Clinical Trials. PLoS Med. 2016;13(4):e1001992. Published 2016 Apr 12. doi:10.1371/journal.pmed.1001992
119. Vinnakota DN, Kamatham R. Safety profile of phentolamine mesylate as reversal agent of pulpal and soft tissue dental anesthesia: a systematic review and meta-analysis. Quintessence Int. 2019;50(7):568-575. doi:10.3290/j.qi.a42574
120. Vukadinović D, Scholz SS, Messerli FH, et al. Peripheral edema and headache associated with amlodipine treatment: a meta-analysis of randomized, placebo-controlled trials. J Hypertens. 2019;37(10):2093-2103. D
121. Wang J, Wang Z, Zhao Y. Incidence and risk of hypertension with ramucirumab in cancer patients: a meta-analysis of published studies. Clin Drug Investig. 2015;35(4):221-228. doi:10.1007/s40261-015-0272-z
122. Wang S, He Q, Shuai Z. Risk of serious infections in biological treatment of patients with ankylosing spondylitis and non-radiographic axial spondyloarthritis: a meta-analysis. Clin Rheumatol. 2018;37(2):439-450. doi:10.1007/s10067-017-3966-1
123. Wang T, Wang F, Zhou J, Tang H, Giovenale S. Adverse effects of incretin-based therapies on major cardiovascular and arrhythmia events: meta-analysis of randomized trials. Diabetes Metab Res Rev. 2016;32(8):843-857. doi:10.1002/dmrr.2804
124. Wang W, Lie P, Guo M, He J. Risk of hepatotoxicity in cancer patients treated with immune checkpoint inhibitors: A systematic review and meta-analysis of published data. Int J Cancer. 2017;141(5):1018-1028. doi:10.1002/ijc.30678
125. Wei A, Gu Z, Li J, et al. Clinical Adverse Effects of Endothelin Receptor Antagonists: Insights From the Meta-Analysis of 4894 Patients From 24 Randomized Double-Blind Placebo-Controlled Clinical Trials. J Am Heart Assoc. 2016;5(11):e003896. Published 2016 Oct 26. doi:10.1161/JAHA.116.003896
126. Wei W, Luo Z. Risk of gastrointestinal toxicities with PD-1 inhibitors in cancer patients: A meta-analysis of randomized clinical trials. Medicine (Baltimore). 2017;96(48):e8931. doi:10.1097/MD.0000000000008931
127. Wessler JD, Giugliano RP. Risk of thrombocytopenia with glycoprotein IIb/IIIa inhibitors across drugs and patient populations: a meta-analysis of 29 large placebo-controlled randomized trials [published correction appears in Eur Heart J Cardiovasc Pharmacother. 2016 Oct;2(4):217]. Eur Heart J Cardiovasc Pharmacother. 2015;1(2):97-106. doi:10.1093/ehjcvp/pvu008
128. Xia N, Wang H, Nie X. Inhaled Long-Acting β2-Agonists Do Not Increase Fatal Cardiovascular Adverse Events in COPD: A Meta-Analysis. PLoS One. 2015;10(9):e0137904. Published 2015 Sep 17. doi:10.1371/journal.pone.0137904
129. Xie W, Huang Y, Xiao S, Sun X, Fan Y, Zhang Z. Impact of Janus kinase inhibitors on risk of cardiovascular events in patients with rheumatoid arthritis: systematic review and meta-analysis of randomised controlled trials. Ann Rheum Dis. 2019;78(8):1048-1054. doi:10.1136/annrheumdis-2018-214846
130. Xing Y, Chen L, Feng Y, Zhou Y, Zhai Y, Lu J. Meta-analysis of the safety of voriconazole in definitive, empirical, and prophylactic therapies for invasive fungal infections. BMC Infect Dis. 2017;17(1):798. Published 2017 Dec 28. doi:10.1186/s12879-017-2913-8
131. Xu H, Tan P, Zheng X, et al. Immune-related adverse events following administration of anti-cytotoxic T-lymphocyte-associated protein-4 drugs: a comprehensive systematic review and meta-analysis. Drug Des Devel Ther. 2019;13:2215-2234. Published 2019 Jul 4. doi:10.2147/DDDT.S196316
132. Xu M, Nie Y, Yang Y, Lu YT, Su Q. Risk of Neurological Toxicities Following the Use of Different Immune Checkpoint Inhibitor Regimens in Solid Tumors: A Systematic Review and Meta-analysis. Neurologist. 2019;24(3):75-83. doi:10.1097/NRL.0000000000000230
133. Yamada A, Wang J, Komaki Y, Komaki F, Micic D, Sakuraba A. Systematic review with meta-analysis: risk of new onset IBD with the use of anti-interleukin-17 agents. Aliment Pharmacol Ther. 2019;50(4):373-385.
134. Yang W, Li S, Yang Q. Risk of dermatologic and mucosal adverse events associated with PD-1/PD-L1 inhibitors in cancer patients: A meta-analysis of randomized controlled trials. Medicine (Baltimore). 2019;98(20):e15731. doi:10.1097/MD.0000000000015731
135. Yang Y, Liu YH, Sun X, et al. Risk of peripheral edema in cancer patients treated with MEK inhibitors: a systematic review and meta-analysis of clinical trials. Curr Med Res Opin. 2017;33(9):1663-1675. doi:10.1080/03007995.2017.1349657
136. Zhang B, Wu Q, Zhou YL, Guo X, Ge J, Fu J. Immune-related adverse events from combination immunotherapy in cancer patients: A comprehensive meta-analysis of randomized controlled trials. Int Immunopharmacol. 2018;63:292-298. doi:10.1016/j.intimp.2018.08.014
137. Zhang H, Huang Z, Zou X, Liu T. Bevacizumab and wound-healing complications: a systematic review and meta-analysis of randomized controlled trials. Oncotarget. 2016;7(50):82473-82481. doi:10.18632/oncotarget.12666
138. Zhang S, Liang F, Li W, Wang Q. Risk of treatment-related mortality in cancer patients treated with ipilimumab: A systematic review and meta-analysis. Eur J Cancer. 2017;83:71-79. doi:10.1016/j.ejca.2017.06.021
139. Zhang S, Liang F, Zhu J, Chen Q. Risk of Pneumonitis Associated with Programmed Cell Death 1 Inhibitors in Cancer Patients: A Meta-analysis. Mol Cancer Ther. 2017;16(8):1588-1595. doi:10.1158/1535-7163.MCT-17-0155
140. Zhang X, Ran Y, Shao Y, Wang K, Zhu Y. Incidence and risk of severe infections associated with aflibercept in cancer patients: a systematic review and meta-analysis. Br J Clin Pharmacol. 2016;81(1):33-40. doi:10.1111/bcp.12758
141. Zhang X, Ran Y, Wang K, Zhu Y, Li J. Incidence and risk of hepatic toxicities with PD-1 inhibitors in cancer patients: a meta-analysis. Drug Des Devel Ther. 2016;10:3153-3161. Published 2016 Sep 28. doi:10.2147/DDDT.S115493.
142. Zhao B, Zhao H, Zhao J. Risk of fatal adverse events in cancer patients treated with sunitinib. Crit Rev Oncol Hematol. 2019;137:115-122. doi:10.1016/j.critrevonc.2019.03.007
143. Zhao YT, Li PY, Zhang JQ, Wang L, Yi Z. Angiotensin II Receptor Blockers and Cancer Risk: A Meta-Analysis of Randomized Controlled Trials. Medicine (Baltimore). 2016;95(18):e3600. doi:10.1097/MD.0000000000003600
144. Zhou JX, Feng LJ, Zhang X. Risk of severe hematologic toxicities in cancer patients treated with PARP inhibitors: a meta-analysis of randomized controlled trials. Drug Des Devel Ther. 2017;11:3009-3017. Published 2017 Oct 13. doi:10.2147/DDDT.S147726
145. Zhou Y, Lu H, Yang M, Xu C. Adverse drug events associated with ibrutinib for the treatment of elderly patients with chronic lymphocytic leukemia: A systematic review and meta-analysis of randomized trials. Medicine (Baltimore). 2019;98(33):e16915. doi:10.1097/MD.0000000000016915
146. Zhu J, Wu J, Li G, et al. Meta-analysis of randomized controlled trials for the incidence and risk of fatal adverse events in cancer patients treated with ipilimumab. Expert Opin Drug Saf. 2017;16(4):423-428. doi:10.1080/14740338.2017.1297420
147. Zhu J, Zhao W, Liang D, et al. Risk of fatigue in cancer patients receiving anti-EGFR monoclonal antibodies: results from a systematic review and meta-analysis of randomized controlled trial. Int J Clin Oncol. 2018;23(2):389-399. doi:10.1007/s10147-017-1218-7
148. Zhu LN, Chen D, Chen T, Xu D, Chen SH, Liu L. The adverse event profile of brivaracetam: A meta-analysis of randomized controlled trials. Seizure. 2017;45:7-16. doi:10.1016/j.seizure.2016.11.008
149. Zhu X, Tian X, Yu C, Hong J, Fang J, Chen H. Increased risk of hemorrhage in metastatic colorectal cancer patients treated with bevacizumab: An updated meta-analysis of 12 randomized controlled trials. Medicine (Baltimore). 2016;95(34):e4232. doi:10.1097/MD.0000000000004232
150. Zhu X, Wu S. Increased Risk of Hypertension with Enzalutamide in Prostate Cancer: A Meta-Analysis. Cancer Invest. 2019;37(9):478-488. doi:10.1080/07357907.2019.1670203
151. 薛晓静,何饶丽,李伟兴,辛佳蔚,叶钦勇,陈晓春,潘晓东.非典型抗精神病药治疗痴呆精神行为症状安全性的系统评价[J].中华医学杂志,2018,98(25):2030-2036.

**Figure S1.** The flow plot.


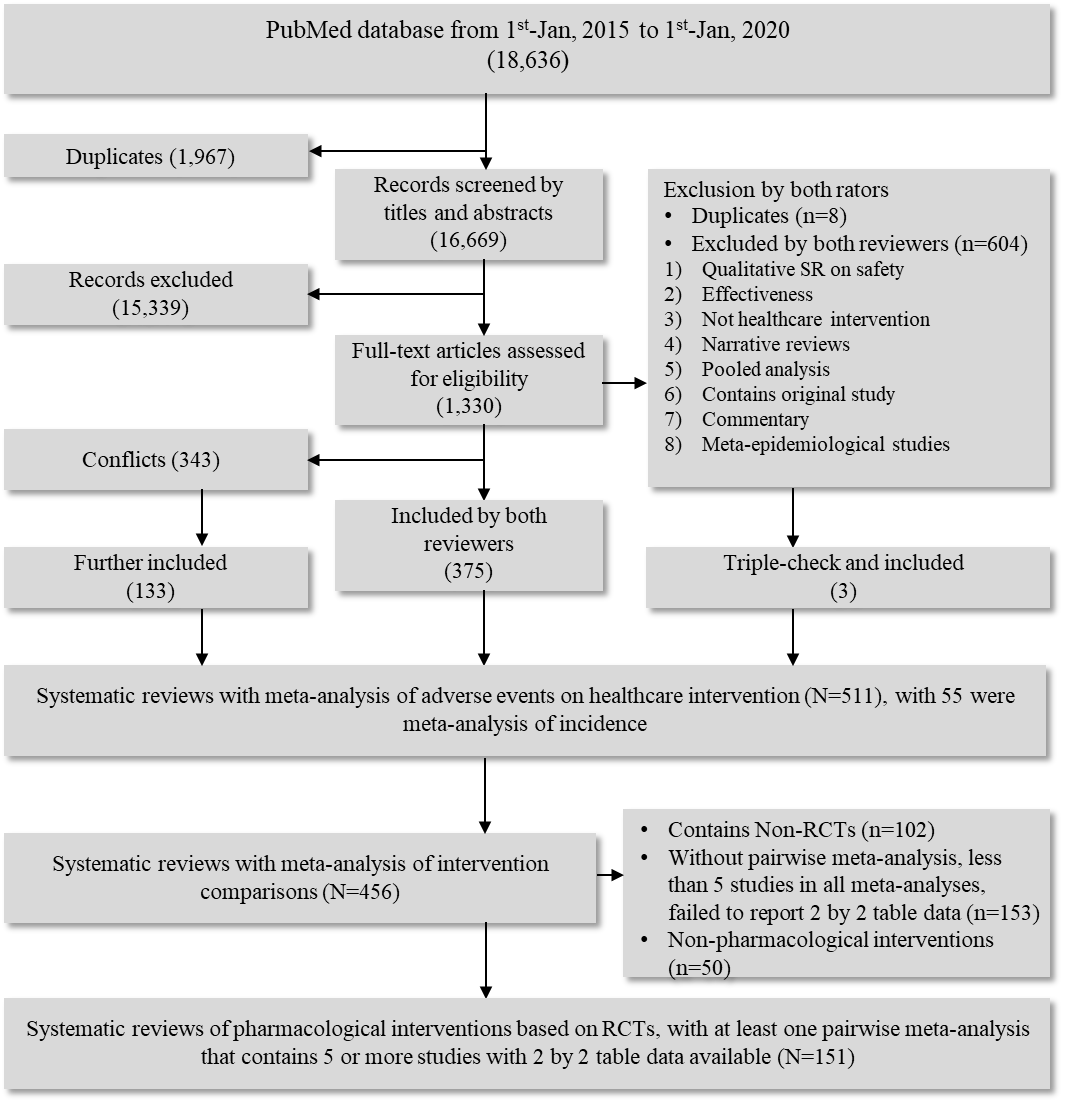


**Figure S2.** Consistency on the point estimates of rapid approach through ClinicalTrials.gov and systematic approach under different events rate.

**
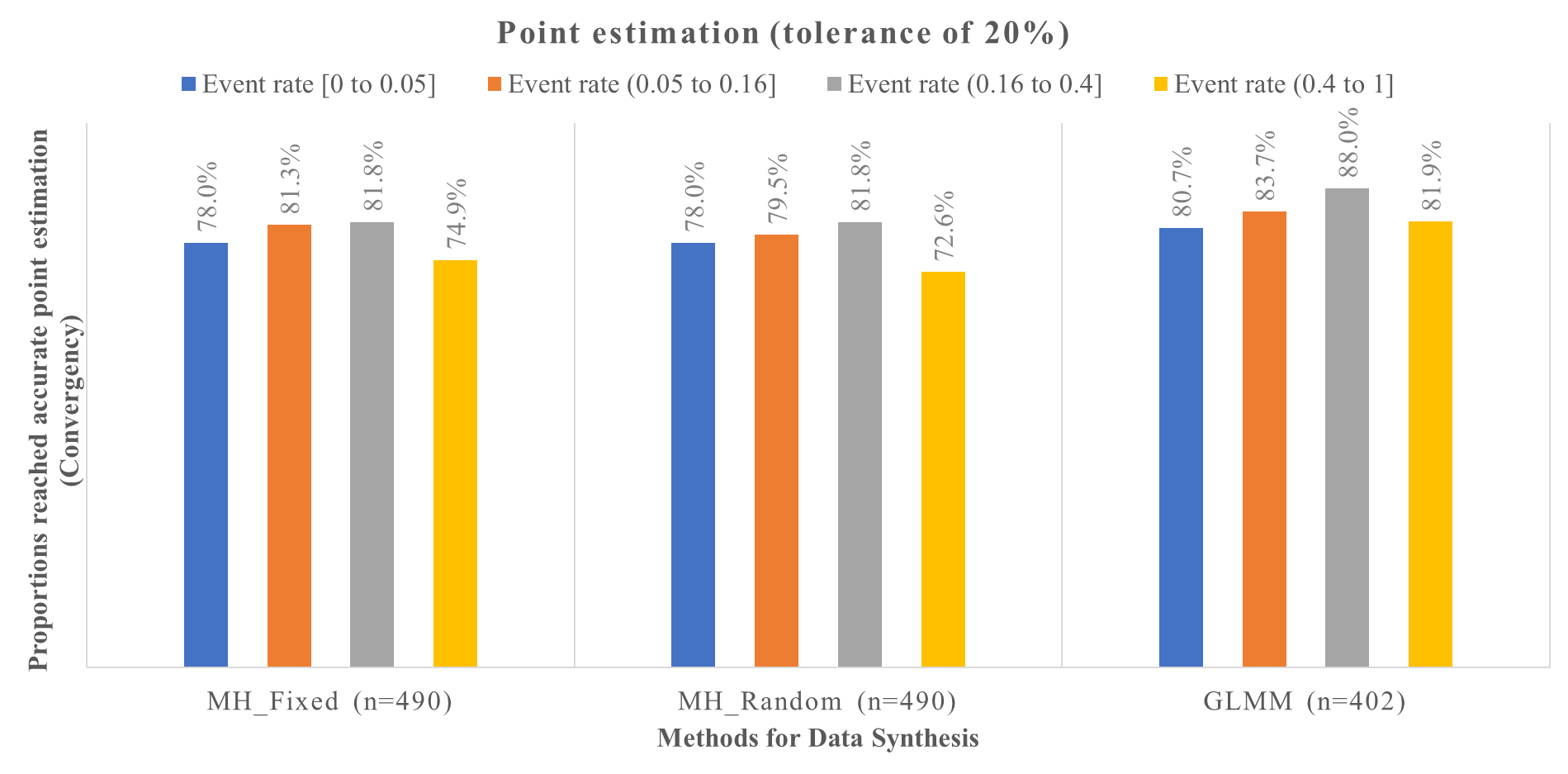
**

**Figure S3.** Consistency on the direction of rapid approach through ClinicalTrials.gov and systematic approach under different events rate.

**
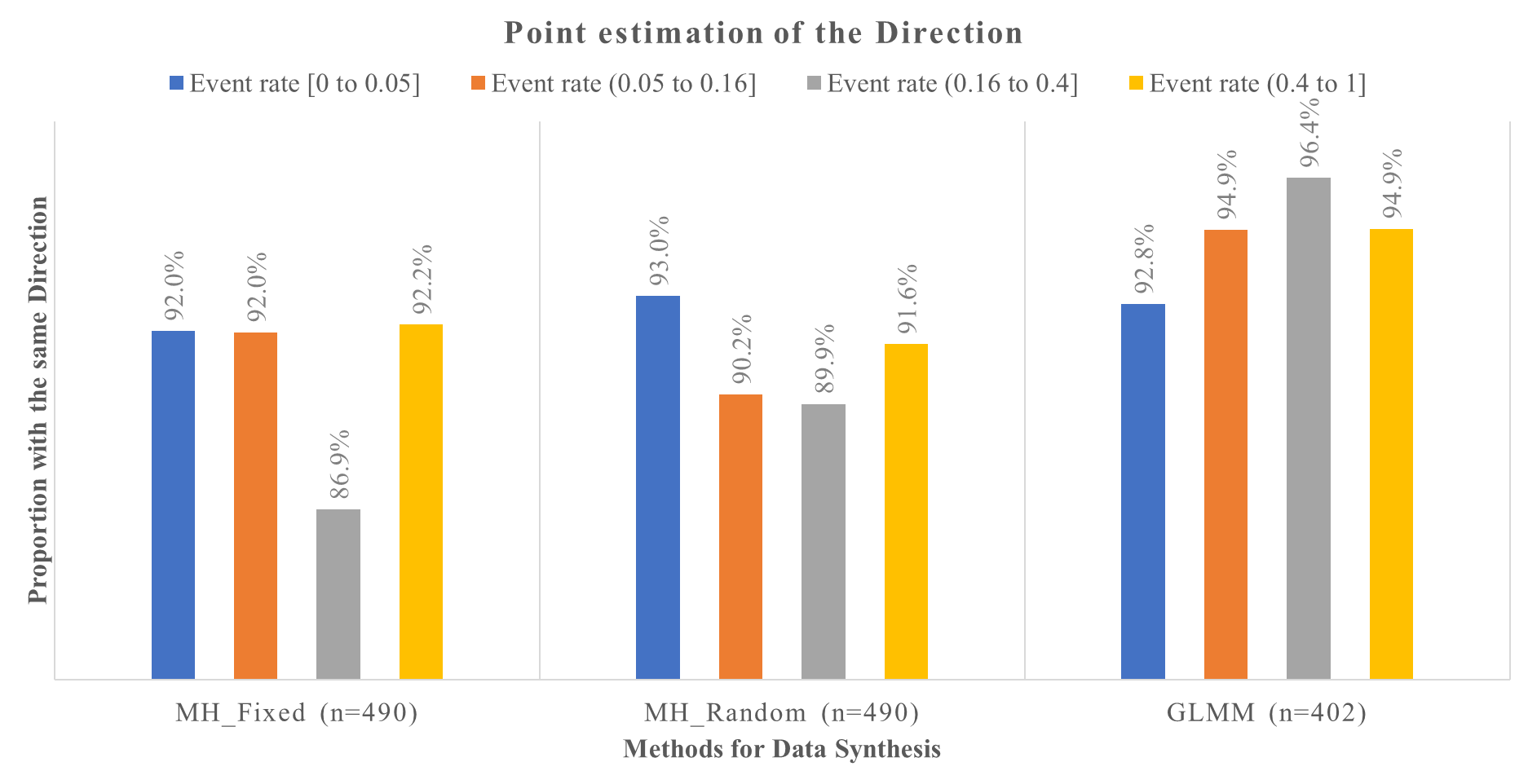
**

**Figure S4.** Consistency on the significance of rapid approach through ClinicalTrials.gov and systematic approach under different events rate.


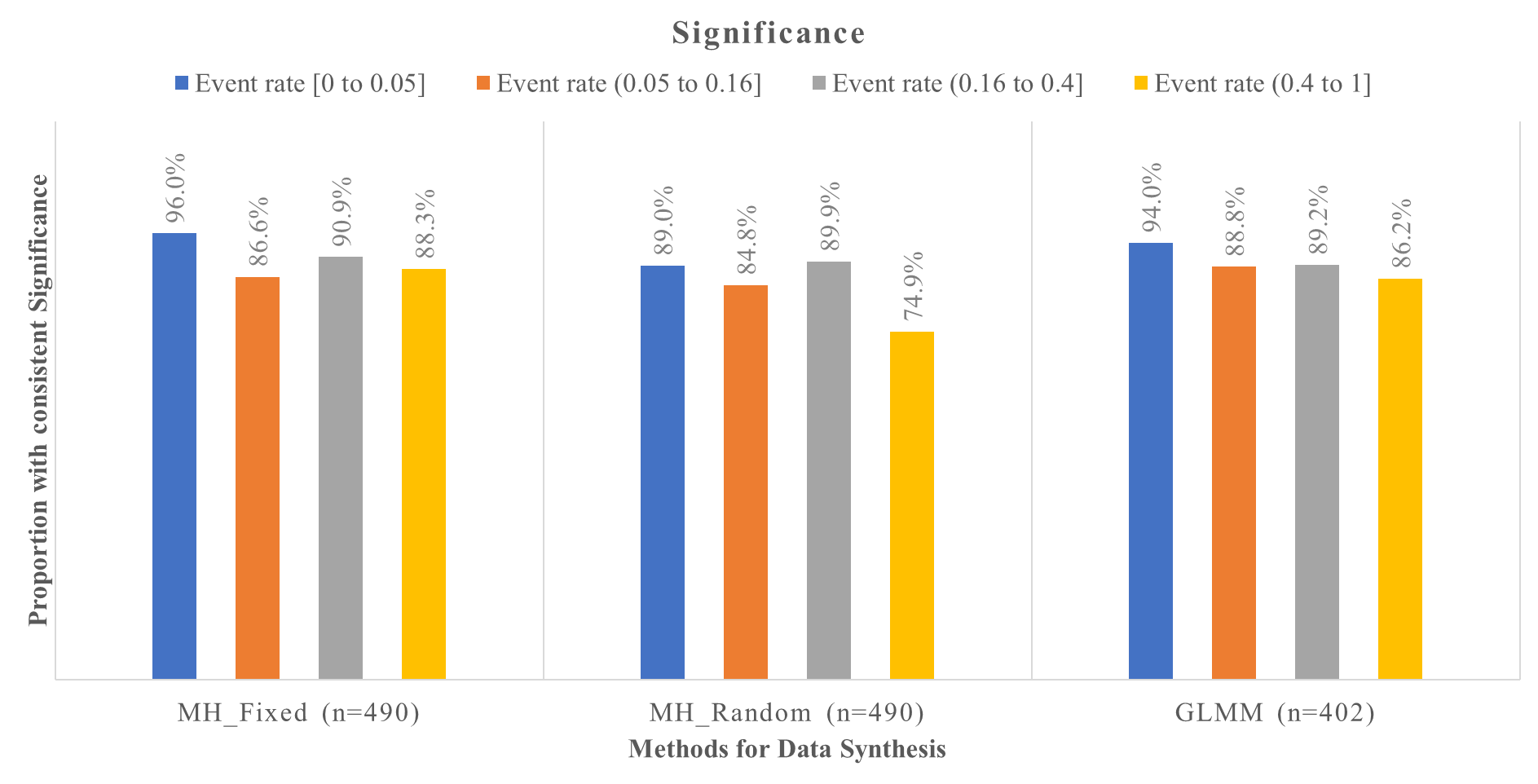


**Figure S5.** Consistency on the point estimates of rapid approach through ClinicalTrials.gov and systematic approach under different amount of heterogeneity.


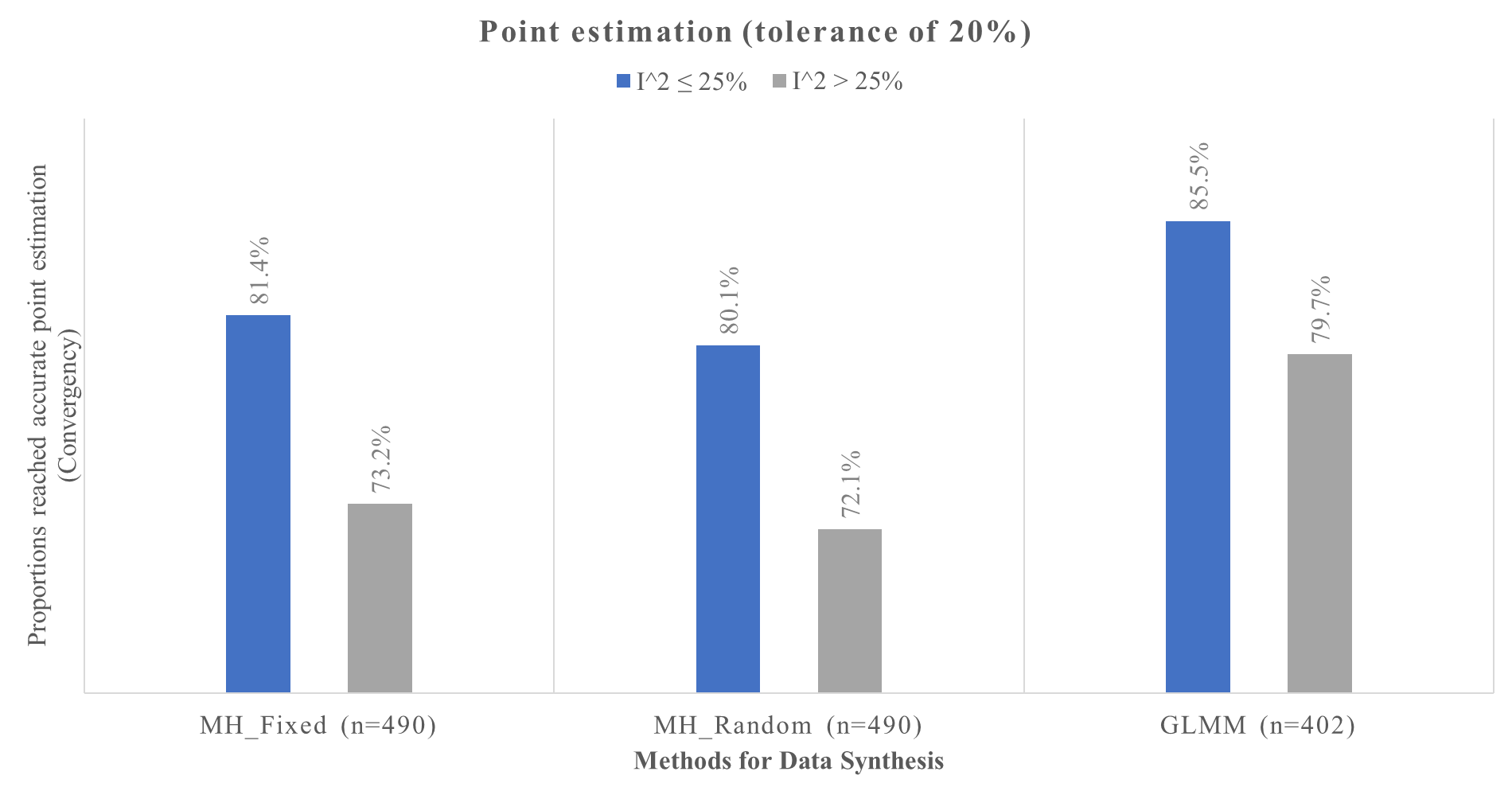


**Figure S6.** Consistency on the direction of rapid approach through ClinicalTrials.gov and systematic approach under different amount of heterogeneity.


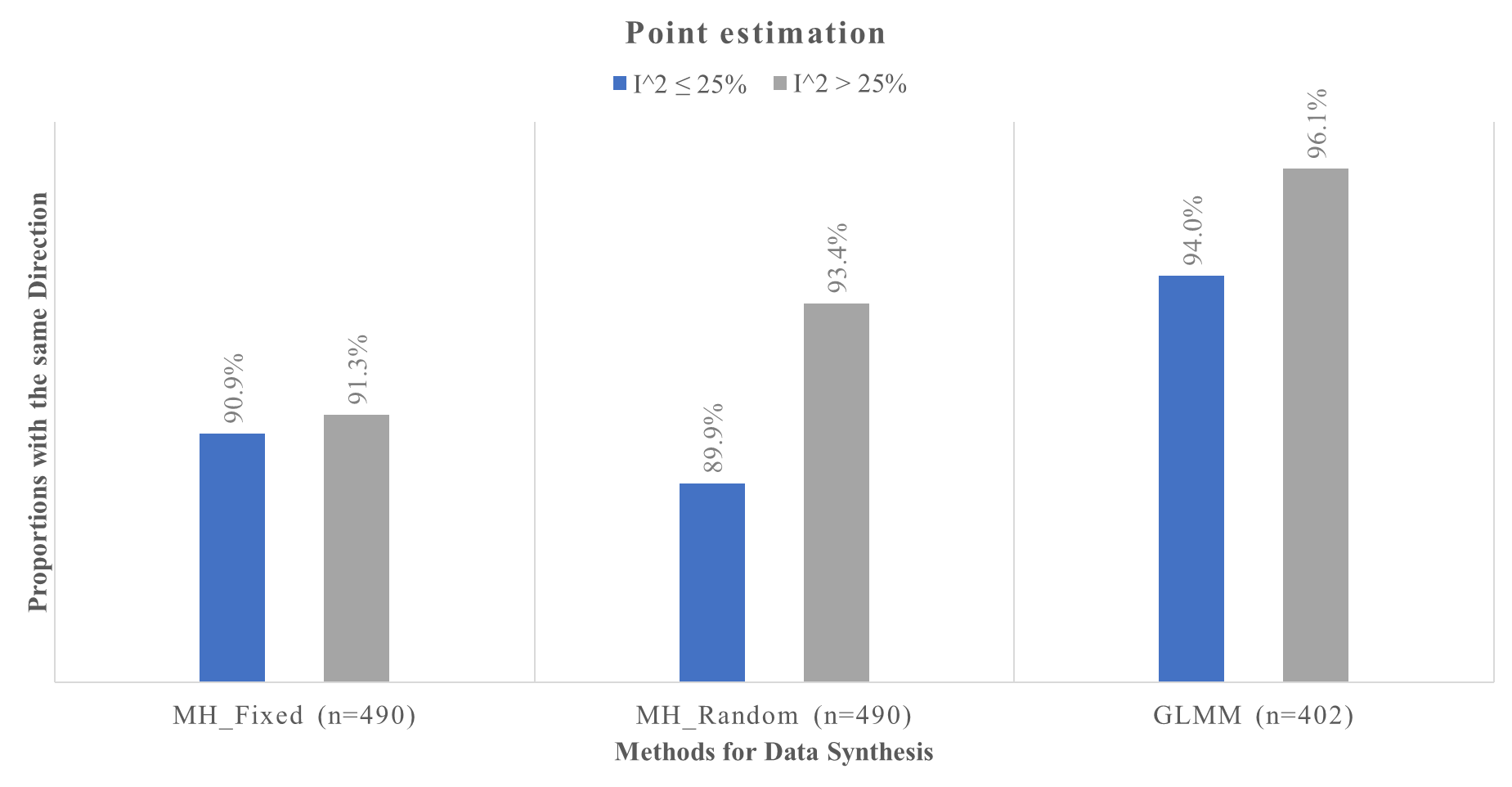


**Figure S7.** Consistency on the significance of rapid approach through ClinicalTrials.gov and systematic approach under different amount of heterogeneity.

**
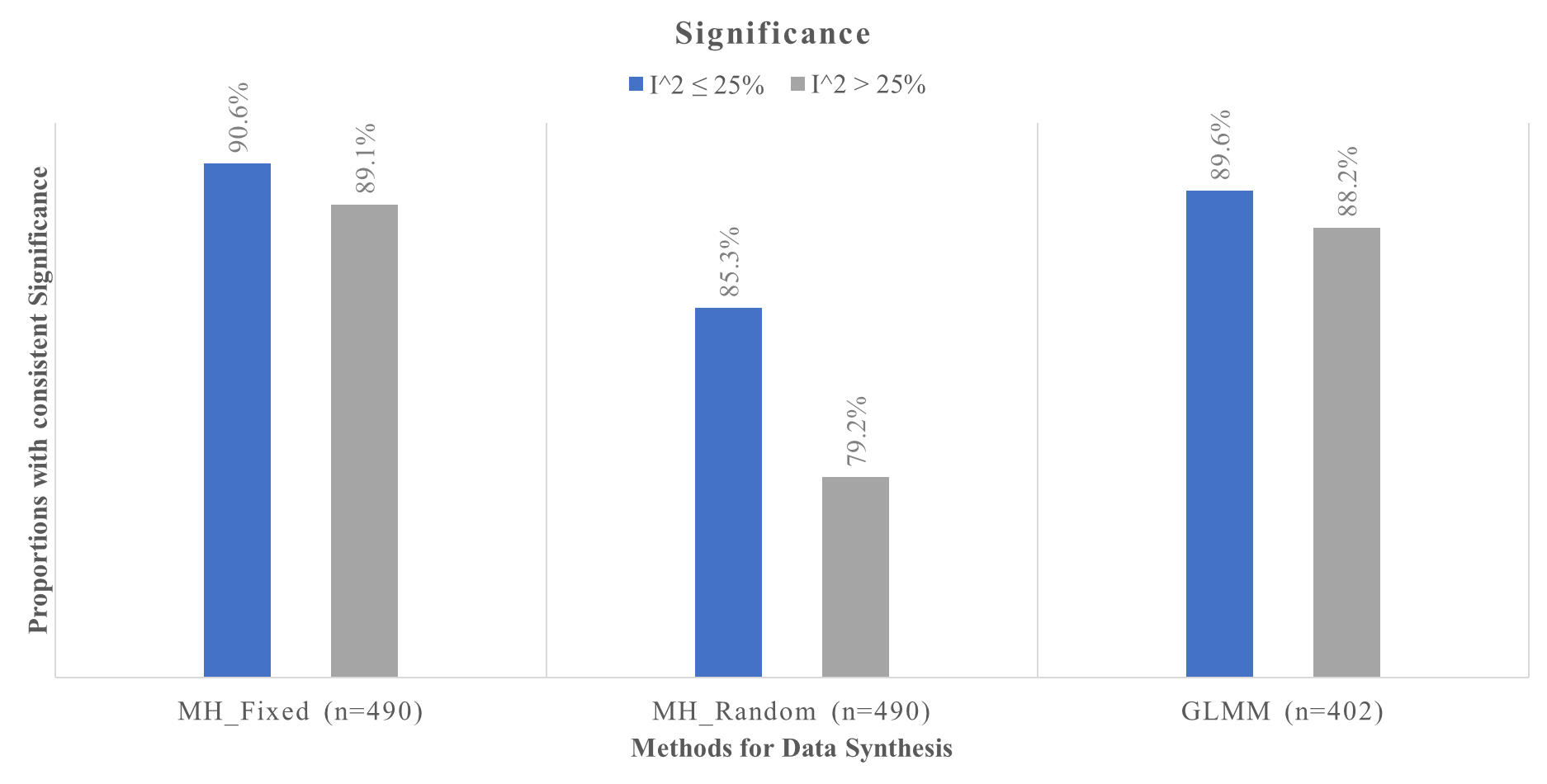
**
